# Nitric Oxide Regulates Estrus Cycle Dependent Colonic Motility in Mice

**DOI:** 10.3389/fnins.2021.647555

**Published:** 2021-09-29

**Authors:** Gayathri K. Balasuriya, Saseema S. Nugapitiya, Elisa L. Hill-Yardin, Joel C. Bornstein

**Affiliations:** ^1^Department of Physiology, The University of Melbourne, Parkville, VIC, Australia; ^2^School of Health and Biomedical Sciences, RMIT University, Bundoora, VIC, Australia; ^3^Faculty of Medicine, The University of Queensland, Herston, QLD, Australia

**Keywords:** estrogen, nitric oxide synthase, enteric neural circuits, colonic motor complexes, ERalpha and ERbeta, immunohistochemistry, neuroplasticity, estrus cycle

## Abstract

Women are more susceptible to functional bowel disorders than men and the severity of their symptoms such as diarrhea, constipation, abdominal pain and bloating changes over the menstrual cycle, suggesting a role for sex hormones in gastrointestinal function. Nitric oxide (NO) is a major inhibitory neurotransmitter in the gut and blockade of nitric oxide synthase (NOS; responsible for NO synthesis) increases colonic motility in male mice *ex vivo*. We assessed the effects of NOS inhibition on colonic motility in female mice using video imaging analysis of colonic motor complexes (CMCs). To understand interactions between NO and estrogen in the gut, we also quantified neuronal NOS and estrogen receptor alpha (ERα)-expressing myenteric neurons in estrus and proestrus female mice using immunofluorescence. Mice in estrus had fewer CMCs under control conditions (6 ± 1 per 15 min, *n* = 22) compared to proestrus (8 ± 1 per 15 min, *n* = 22, One-way ANOVA, *p* = 0.041). During proestrus, the NOS antagonist N-nitro-L-arginine (NOLA) increased CMC numbers compared to controls (189 ± 46%). In contrast, NOLA had no significant effect on CMC numbers during estrus. During estrus, we observed more NOS-expressing myenteric neurons (48 ± 2%) than during proestrus (39 ± 1%, *n* = 3, *p* = 0.035). Increased nuclear expression of ERα was observed in estrus which coincided with an altered motility response to NOLA in contrast with proestrus when ERα was largely cytoplasmic. In conclusion, we confirm a cyclic and sexually dimorphic effect of NOS activity in female mouse colon, which could be due to genomic effects of estrogens via ERα.

## Introduction

Sexual dimorphism in gastrointestinal (GI) disorders is well documented and includes a higher prevalence of visceral pain disorders such as irritable bowel syndrome and gastroesophageal reflux disease as well as gallbladder and biliary tract diseases in women than in men of a similar age and health status (Palomba et al., 2011; Avramidou et al., 2018; Kim and Kim, 2018). Fluctuations in female hormone levels during the menstrual cycle, pregnancy, premenopausal, or menopausal states are associated with nausea, vomiting, abdominal pain, distension, abnormal satiety, bloating, diarrhea, or constipation (Hutson et al., 1989; Mayer et al., 1999). During pregnancy, constipation is increased and gastrointestinal motility is reduced (Tytgat et al., 2003; Bradley et al., 2007; Cullen and O’Donoghue, 2007; Boregowda and Shehata, 2013). However, the mechanisms underlying differential effects on GI motility in females are not well studied.

Rodents have a 4-day estrus cycle that includes fluctuations of sex hormones and are therefore a useful preclinical model for investigating influences of sex-hormones on GI function. The estrus cycle has four distinct stages, including proestrus, estrus, metestrus, and diestrus. During proestrus, which lasts for 18–24 h, plasma estrogen levels are high and fall drastically during the estrus phase resulting in ovulation. In contrast, primates (including human females) are in a 28-day menstrual cycle. Plasma estrogen levels reach a peak toward the end of the human follicular phase (comparable with the proestrus stage in rodents) and drop during ovulation which marks the start of the luteal phase (comparable with the estrus stage in rodents). During the diestrus phase, female rats have lower estrogen levels and slower gastric emptying than during estrous/proestrus (Chen et al., 1995). Estrogen also inhibits Cl^–^ secretion across the human and rat distal colon epithelium (O’Mahony et al., 2007; O’Mahony and Harvey, 2008). Estrogen dose-dependently inhibited the secretory responses induced by a well-established secretagogue cholera toxin in female rats, an effect seen in estrus but not in proestrus, but had no inhibitory effect on male rats (Alzamora et al., 2011a,b). Estrogen receptors are expressed on the cell membrane and in the nucleus both in mucosal epithelial cells and in enteric neurons of rodent gut (Campbell-Thompson et al., 2001; Li et al., 2016; Liu et al., 2019). Through these receptors, estrogen reportedly influences rodent gut physiology including secretion and motility (O’Mahony et al., 2007; Alzamora et al., 2011a; Li et al., 2016; Liu et al., 2019). For example, gastric emptying is significantly delayed in pregnant female rats (Shah et al., 2000, 2001). In addition, administration of the circulating form of estrogen, estradiol (alone or together with progesterone) reduced gastric emptying in adult female rats (Chen et al., 1995). A recent study, indeed, suggests that estrogen, not progesterone, is responsible for reducing rapid gastric emptying and restoring gastric nitrergic relaxation in ovariectomized mice fed with a high fat diet (Sprouse et al., 2020).

In women, estrogen concentrations are reported to correlate with the menstrual cycle dependent changes in amplitude and velocity of the jejunal migrating motor complex (Aytuğ et al., 2001). Moreover, several lines of evidence suggest that changes in rodent gut function during the estrus cycle are primarily due to fluctuations in estrogen rather than progesterone. Colonic transit is slower during the proestrus-estrus phases and faster during the metestrus-diestrus phases in female rats (Chen et al., 1995). In the absence of the estrus cycle (i.e., in ovariectomized female rats), colonic transit is faster in keeping with a metestrus-diestrus phenotype typical of low estrogen levels (Ryan and Bhojwani, 1986). The literature also suggests that the A-type potassium currents that regulate neuronal excitability are suppressed by estrogen and could be linked to altered colonic motility associated with menstrual cycle, pregnancy, and menopause (Beckett et al., 2006). Furthermore, estrogens modulate colonic function in mice via G protein-coupled estrogen receptors (GPER) expressed in gut neurons (Li et al., 2016) and ileal contractility via actions on GPER and the estrogen receptors alpha (ERα) and beta (ERβ), both of which are expressed in enteric neurons (Liu et al., 2019).

The enteric neural circuitry regulating colonic motility contains sensory neurons, interneurons and (excitatory and inhibitory) motor neurons. These neurons use different neurotransmitters including acetylcholine (ACh), vasoactive intestinal peptide (VIP), serotonin (5-HT), and the main inhibitory neurotransmitter in the colon; nitric oxide (NO; Furness, 2006). Neuronal nitric oxide synthase (nNOS) catalyzes the *de novo* synthesis of NO and is expressed by both inhibitory motor neurons that innervate the circular muscle layer of the mouse colon (Sang and Young, 1996; Spencer et al., 1998) and some myenteric interneurons (Sang and Young, 1996). Colonic contraction complexes include motility patterns that are neurally regulated, rhythmic and spontaneously occurring known as colonic motor complexes or CMCs (Spencer, 2001; Corsetti et al., 2019).

Estrogen potentiates NOS activity in the brain and in the enteric nervous system (ENS; Weiner et al., 1994; Figueroa and Massmann, 1995; Kleinert et al., 1998; McNeill et al., 2002). Interactions between NO activity and the estrus cycle in rodent brain tissue are well documented (Collado et al., 2003; Carrillo et al., 2007; Sica et al., 2009). In the GI tract, estrogens enhance nNOS protein expression in myenteric neurons of the gastric fundus and the colon of estrogen-treated ovariectomized female rats (Shah et al., 2000, 2001). Chronic estrogen deficiency (in follicle stimulating hormone receptor knock-out female mice) decreases nNOS activity leading to reduced nitrergic relaxation in the gastric antrum and also contributes to reduced motility observed in gastroparesis (Ravella et al., 2013). It remains unclear, however, whether changes in estrogen levels affect colonic activity via the inhibitory neurotransmitter, NO. In this study we therefore investigated the role of NO in cyclic differences using *ex vivo* colonic motility and immunofluorescence assays during low (estrus) and high (proestrus) estrogen phases in female mice.

## Materials and Methods

### Animal Tissue

Eight to twelve-week-old female mice in estrus and proestrus were selected by scanning vaginal smears (Tran et al., 2012; Cora et al., 2015; Balasuriya et al., 2016). Briefly, vaginal content was gently flushed into a blunted sterile Pasteur pipette containing 30–50 μl of distilled water and contents were air dried on a microscope slide and stained using a Shandon Wright-Giemsa Stain Kit (Thermo Fisher Scientific, Australia). Slides were scanned with a light microscope (AxioCam, Carl Zeiss Pty., Ltd., North Ryde, NSW, Australia) and proestrus and estrus were identified by their specific cytological profiles. As per (Balasuriya et al., 2016), proestrus cytology consisted of large numbers of nucleated epithelial cells whereas cells from estrus stage vaginal smears were cornified, non-nucleated epithelial cells. Female mice were maintained under a 12 h/12 h light-dark cycle from 0600 to 1800 h. Vaginal smears were taken between 0800 and 0900 h. Animals were culled by cervical dislocation around 1000 h, as approved by the University of Melbourne Animal Experimentation Ethics Committee (approval no: 1011897) and according to guidelines of the National Health and Medical Research Council of Australia. The whole colon was dissected and placed in an organ bath superfused with warmed (36°C) physiological saline solution (composition, mM: NaCl 118, KCl 4.6, NaH_2_PO_4_ 1, NaHCO_3_ 25, MgSO_4_ 1.2, D-glucose 11, CaCl_2_ 2.5; bubbled with 95% O_2_–5% CO_2_). The oral end of the colon tissue was cannulated to an inlet connected to a reservoir of physiological saline, and the anal end to an outflow tube that provided a back-pressure of 3–4 cm H_2_O (Gwynne et al., 2004; Roberts et al., 2007; Swaminathan et al., 2016).

### Video Imaging

**FIGURE 1:**
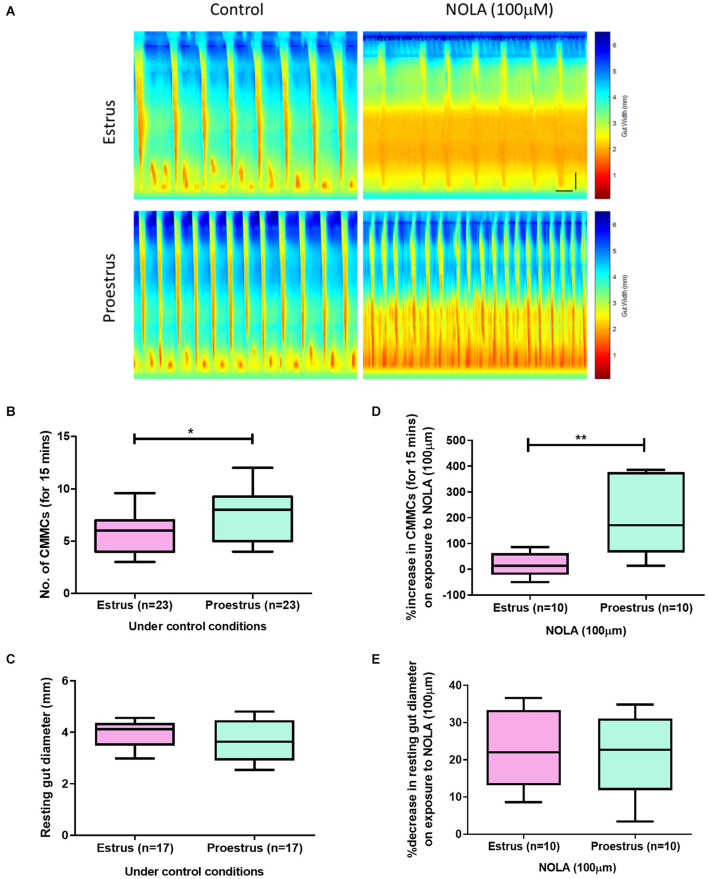
**(A)** Spatiotemporal maps showing the number of CMCs during 15 min recordings of estrus and proestrus colon under the control conditions and on exposure to NOLA (100 μM). **(B)** Females in estrus had fewer CMCs compared to proestrus (*n* = 23, unpaired *t*-test, *p* = 0.042). **(C)** Resting colonic diameters were similar between the groups under control conditions. **(D)** Proestrus colons showed a larger percentage increase in the number of CMCs on exposure to NOLA compared to colons from mice during estrus (*n* = 10, unpaired *t*-test, *p* = 0.003). **(E)** NOLA similarly decreased resting gut diameter in both estrus and proestrus groups (*n* = 10, unpaired *t*-test, *p* = 0.97). Horizontal scale bar = 60 s, vertical scale bar = 0.5 cm. **p < 0.01 and *p < 0.05.

Video imaging analysis of colonic motility was conducted as previously described (Balasuriya et al., 2016; Swaminathan et al., 2016) using female mice during estrus and proestrus. Briefly, colonic motility was recorded *ex vivo* using a Logitech camera (QuickCam Pro 4000; I-Tech, Ultimo, NSW, Australia) mounted directly above the organ bath. In-house software (Scribble 2.0) and a purpose-built (Matlab, 2014) plugin, Analyse 2.0, were used to convert recorded video segments (15 min duration) to spatiotemporal maps where the diameter of the colon is mapped [as a spatiotemporal (ST) map] along the length of the segment as a function of time (*x* axis: time, *y* axis: colon length). Blue–green pixels on ST maps indicate relaxed tissue and yellow–red pixels identify constricted regions (Figure 1). Under the experimental conditions of 2–4 cm H_2_O pressure migrating motor complexes originating at the oral end and propagating to anal direction were observed in the colon preparations. The frequency of propagating contractions previously described as colonic migrating motor complexes (CMMCs) was manually counted from the ST maps (Balasuriya et al., 2016). Resting colonic diameter measures were obtained using in built functions of the software as previously reported (Gwynne et al., 2004; Balasuriya et al., 2016).

Motility experiments began with a 30 min equilibration period, followed by four 15 min control video recordings (1 h control period). The effects of NOS inhibition were studied by adding the NOS antagonist Nitro-L-arginine (NOLA; 100 μM, Sigma-Aldrich, St. Louis, MO, United States, Middlesex, United Kingdom) to the bath. Motility was recorded for another 1 h (i.e., in the form of four 15 min video files). A 1 h washout period during which the drug solution was replaced with physiological saline was subsequently conducted.

### Immunohistochemistry

#### Tissue Preparation

The immunofluorescence method used in this study was as previously reported (Neal and Bornstein, 2007; Roberts et al., 2007) with minor adaptations. Briefly, colons were dissected from the culled mice (males, females during estrus, and proestrus) and placed in a petri dish lined with a silicon elastomer (Sylgard 184, Dow Corning, United States) containing phosphate buffered saline (0.1 M PBS, pH 7.2, filtered three times). The colon was cut open along the mesenteric boarder and stretched and pinned flat (mucosal side facing up) onto the petri dish using insect pins (supplier, Sydney, NSW, Australia). Tissue preparations were fixed in 4% formaldehyde overnight at 4°C. The fixative was then washed (three 10 min washes in 0.1 M PBS). Using fine dissecting instruments, the mucosa, submucosal plexus and circular muscle layers were carefully peeled away from the distal colon to expose the myenteric neurons with an underlying longitudinal muscle layer (LMMP). This whole mount preparation was used for immunofluorescence. A small number of circular muscle cells remained following peeling of the mucosa and SMP layers to reveal myenteric ganglia.

#### Immunofluorescence

Whole mount preparations of myenteric plexus were permeabilized and blocked using 0.01% triton (ProSciTech, Thuringowa Central, QLD, Australia) in 10% CAS block (Invitrogen Australia Pty., Ltd., Mount Waverley, VIC, Australia) for 30 min at room temperature and incubated overnight with primary antisera (Table 1) at 4°C. The next day, tissue samples were washed three times at 10 min intervals using filtered 0.1 M PBS and incubated with fluorescence tagged secondary antisera (Table 1) for 2.5 h at room temperature. Preparations were then washed with filtered PBS and mounted on slides using fluorescence mounting medium (Dako Australia Pty., Ltd., Botany, NSW, Australia) and secured with a glass coverslip. Images were captured using a confocal microscope (Zeiss LSM510 fluorescence microscope; Zeiss, Gladesville, NSW, Australia) and Zeiss LSM software, (version 4.2.0.121).

**TABLE 1:** Antisera used for evaluation of NOS neurons and estrogen receptor alpha (ERα) in whole mount preparations of myenteric plexus.

Antisera	Source	Dilution
Sheep anti NOS	Gift from Dr. P. Emson #K205	1:1000
Human anti Hu	Gift from Dr. V. Lennon	1:10000
Rabbit anti ERα	Thermo Fisher Scientific #PA1 308	1:400
Rabbit anti ERβ	Abcam #ab3577	1:300
Donkey anti sheep Alexa 488	Molecular Probes # A11615	1:1000
Donkey anti Human Alexa 594	Molecular Probes # 709-585-149	1:1000
Donkey anti Rabbit Alexa 647	Molecular Probes # A31573	1:2000

Neurons were considered to be part of a separate ganglion if they were separated from the edge of the nearest neuron by at least two cell body widths as described by Baudry et al. (2012). Using that definition as a guide, six randomly selected ganglia from each region of the colon were counted. The neuronal marker Hu (anti-ANNA-1, 1:10000 dilution, a gift from Dr. Lennon, United States) was used to label all the neurons of the preparation (Hotta et al., 2013; Fung et al., 2014). The number of Hu positive cells corresponded to the total number of neurons in counted ganglia. Numbers of ERα and ERβ positive cells per ganglion as well as the expression in the nucleus were manually counted using ImageJ software and its Cell counter plugin (NIH, Bethesda, MD, United States) and the percentage was calculated.

### Statistical Analyses

Data were analyzed by one-way ANOVA or Student’s *t* test as appropriate. n is the number of animals from which measures were taken, and statistical significance was set at *P* < 0.05. Data are presented as mean ± standard error of the mean (SEM).

## Results

### Reduced Number of CMCs During Estrus Compared to Proestrus

To assess differences in GI motility between estrus and proestrus, we examined CMC parameters in female mice under control conditions. An advantage of using the *ex vivo* organ bath approach is that it enables large intestinal motility to be studied without the influence of the central nervous system. Mice in estrus exhibited fewer CMCs compared to proestrus (estrus: 6 ± 1 CMCs per 15 min, *n* = 23; proestrus: 8 ± 1 CMMCs/15 min, *n* = 23; *p* = 0.041, Figures 1A,B). However, resting colonic diameter was unchanged between estrus and proestrus (3.9 ± 0.14 mm and 3.7 ± 0.21 mm, respectively, *n* = 17 in each group; *p* = 0.326, Figure 1C).

### NOLA Exerts an Estrus Cycle Dependent Effect on CMC Frequency

It is well established that CMC frequency is increased in the presence of the NOS inhibitor, NOLA, in male mouse colon (Spencer et al., 1998; Spencer, 2001). When responses of estrus and proestrus colons to NOLA were compared (Figure 1D), proestrus colon preparations had a greater percentage increase in CMC numbers (189 ± 46%) than estrus colons (15 ± 14%, *p* = 0.0028, *n* = 10 in each group). Control values were 7 ± 1 and 5 ± 1 CMCs/15 min for estrus and proestrus, respectively, and 7 ± 1 and 9 ± 1 CMCs/15 min for estrus and proestrus, respectively, under NOLA exposure (*n* = 10).

In the same preparations, NOLA significantly reduced the resting colonic diameter in estrus and proestrus compared to control conditions (estrus: NOLA 2.7 ± 0.1 mm, control 3.5 ± 0.03 mm, *p* = 0.01; proestrus: NOLA 2.7 ± 0.1 mm, control 3.2 ± 0.1 mm, *p* = 0.001). But the percentage decrease in resting colonic diameter between estrus and proestrus colons in response to NOLA was unchanged (23 ± 3% and 22 ± 3%, respectively, *p* = 0.97, Figure 1E). Contraction velocity and the distance over which contractions propagate were also unchanged.

### Increased Numbers of NOS Immunoreactive Myenteric Neurons in Estrus

**FIGURE 2:**
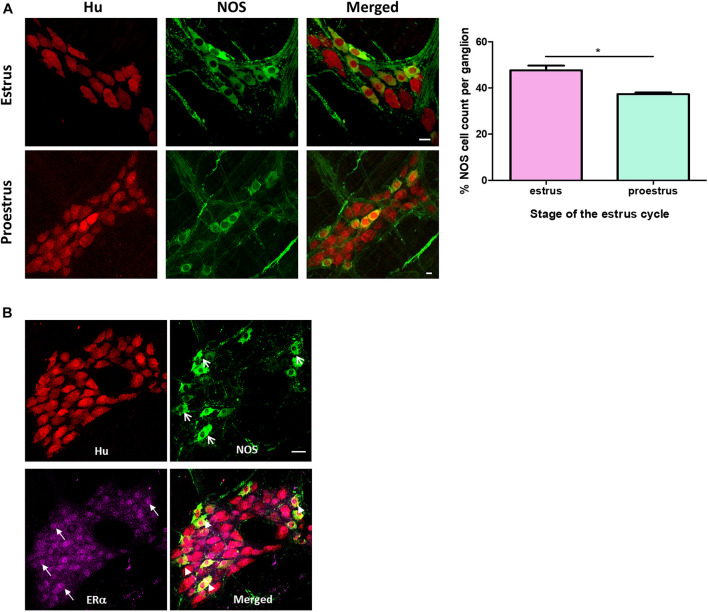
**(A)** Immunofluorescence histochemical double-staining of the whole-mount preparations of the mouse colon. Estrus myenteric neurons had a significantly greater number of NOS expressing neurons compared to proestrus myenteric plexus (estrus 48 ± 2% and proestrus 39 ± 1%, *n* = 3, *p* = 042 one-way ANOVA). The pan neuronal marker Hu is shown in red and NOS expressing myenteric neurons in green. Scale bar = 20 μm. **(B)** Co-labeling of NOS neurons with estrogen receptor alpha in a randomly selected female mouse. Whole mounts of myenteric ganglia showing co-labeling of Hu (red), NOS (green), and ERα (magenta). ERα is expressed in the majority of nuclei of myenteric neurons (closed arrows). Arrowheads indicate myenteric neurons co-labeled with NOS and ERα. Scale bar = 30 μm. *p < 0.05.

A greater proportion of myenteric neurons were immunoreactive for nNOS in distal colons from mice in estrus compared with proestrus. During estrus, 48 ± 2% of the total number of myenteric neurons per ganglion were immunoreactive for nNOS (215 of 457 Hu-positive myenteric neurons expressed NOS; *n* = 3 animals, Figure 2A). During proestrus, 39 ± 1% of myenteric neurons expressed nNOS (160 neurons of a total of 414 Hu-positive myenteric neurons expressed NOS; *n* = 3 animals; *p* = 0.042, Figure 2A). nNOS labeling was also observed in myenteric varicosities and in varicosities adjacent to residual smooth muscle cells present in the myenteric plexus preparations.

### Prominent Nuclear Expression of ERα During Estrus

**FIGURE 3:**
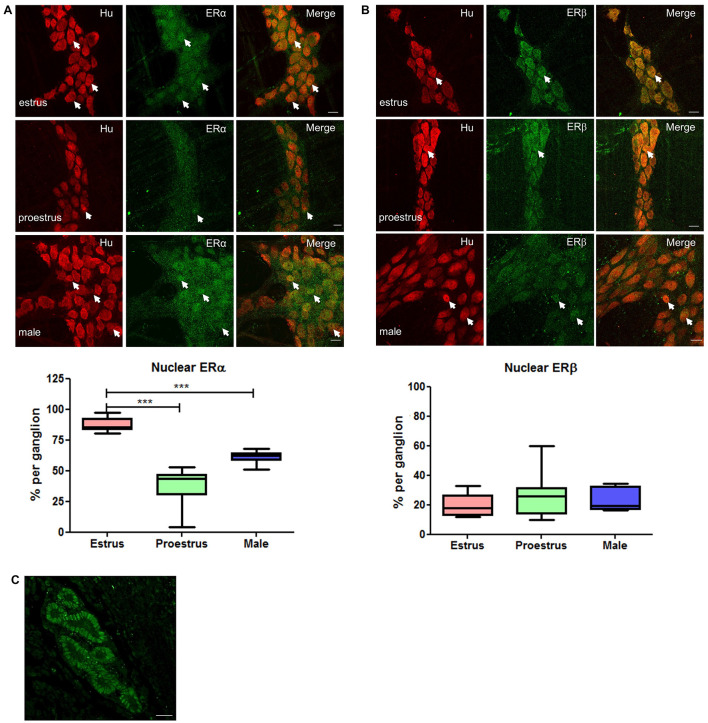
Whole mount preparations of myenteric plexus expressing pan neuronal marker Hu in red and ERα/ERβ in green. Significantly higher numbers of myenteric neurons expressed ERα in the nucleus during estrus while having some cytoplasmic expression compared to proestrus females and male myenteric neurons expressing less nuclear and cytoplasmic ERα. ERβ expression was more cytoplasmic in all tissue samples and showed no significant difference in expression between groups. Scale bar = 20 μm (*n* = 3, One-way ANOVA, ***p < 0.001). **(A)** Localization of ERα in the myenteric plexus preparations of estrus, proestrus, and male colon: ERα labeling was observed in more than 90% of myenteric neurons in both male and female mice. Images of representative myenteric ganglia, demonstrating neurons stained for Hu, ERα, and merged images of both Hu and ERα. During estrus stage, ERα labeling is predominantly nuclear while during proestrus and in male mice both nuclear and cytoplasmic labeling was observed (arrows indicate nuclear labeling of ERα). Scale bars = 20 μm. Graph shows quantified data (*n* = 3) where there was significantly higher nuclear localization observed during estrus stage compared to both proestrus and male preparations (***p < 0.001). **(B)** Localization of ERβ in the myenteric plexus preparations of estrus, proestrus, and male colon: localization of the estrogen receptor beta (ERβ) is unchanged during estrus. ERβ labeling was observed in more than 90% of myenteric neurons in both male and female mice. Images of representative myenteric ganglia, demonstrating neurons stained for Hu, ERα, and merged images of Hu and ERα. No significant difference was observed among the groups with respect to nuclear labeling of ERβ (arrows indicate nuclear labeling of ERβ). Scale bars = 20 μm. The graph shows quantified data (*n* = 3) where there was no significant difference among the groups (*p* > 0.05). **(C)** Specificity of ERα PA1-308-antiserum was tested in a uterine tissue. Labeling was observed in the glandular epithelial cells of the uterus (frozen cross section). Scale bar = 20 μm.

To investigate how motility might be altered with changes in estrogen concentrations during the estrus cycle, we used immunohistochemistry to localize estrogen receptors in myenteric plexus in male and female mice. The ERα antibody was first tested in cross sections of uterine tissue and clear labeling of ERα was observed in the glandular epithelial cells (Figure 3C). Labeling for estrogen receptors in female mice in estrus (*n* = 3) and proestrus (*n* = 3) periods was conducted. ERα labeling was observed in 87 ± 2% and 90 ± 3% of Hu positive myenteric neurons in males and females, respectively (Figure 3A). ERα was strongly labeled in neuronal cell bodies, but some labeling was also present in neuronal processes. In some preparations, low levels of ERα labeling were observed in residual circular muscle as reported previously (Beckett et al., 2006). During estrus, ERα is predominantly localized within the nucleus. In contrast, during proestrus ERα labeling was observed in the cytoplasm of most neurons, with few neurons showing a nuclear localization. The distribution of ERα in males was more uniform with labeling in both the nuclei and cytoplasm of most neurons in the myenteric plexus. During estrus, the proportion of neurons labeling for Hu together with ERα in the nucleus was greater compared to both proestrus females and males (87 ± 2.03, 42 ± 1.51, and 61 ± 1.44, respectively, *p* < 0.001, Figure 3A).

Using the approach described above, we quantified the subcellular localization of the ERβ antiserum (obtained from Prof. John Hutson, Surgical Research Group, Infection and Immunity, Murdoch Children’s Research Institute, Parkville, VIC, Australia; Nation et al., 2011) in mouse myenteric plexus in the colon. In contrast with the findings for ERα, we found no differences in the nuclear localization of ERβ in the myenteric plexus between estrus or proestrus stages or in males (20 ± 3, 27 ± 6, and 23 ± 3, respectively, Figure 3B).

We observed ERα expression in all NOS immunoreactive myenteric neurons as well as in nearly all nNOS-negative neurons (Figure 2B). Thus, the biological machinery required for estrogen to exert a transcriptional effect on neurons regulating colonic motility is present in mouse myenteric neurons.

## Discussion

In this study we showed that altered colonic motility during the estrus cycle correlates with fluctuating levels of sex steroid hormones in female C57Bl/6 mice. Estrogen levels regulate the synthesis of NO (Weiner et al., 1994; Grohe et al., 2004; Upmacis et al., 2011), a potent inhibitory neurotransmitter in the gut affecting motility (Spencer et al., 1998; Spencer, 2001; Powell and Bywater, 2001). During estrus, when plasma estrogen concentrations are low, there were fewer CMCs compared to proestrus when plasma concentrations are high. It is well established that many functional effects of estrogens on gene expression are elicited within the cell nuclei (Serova et al., 2006). Although plasma estrogen concentrations are higher during proestrus, the genomic effects of estrogens that influence secretion in the GI tract of rodents do not occur until estrus (Alzamora et al., 2011a,b). This delay in effect could be due to the time taken for translocation of estrogen-estrogen receptor complexes to cell nuclei and the time course of protein synthesis.

We found ERα expression in almost all Hu positive, and particularly in all nNOS immunoreactive, myenteric neurons. Changes in localization and function of estrogen receptors are observed with altered neuronal functioning in both the CNS (McEwen and Alves, 1999; Maggi et al., 2004; Hedges et al., 2012) and the ENS (Campbell-Thompson et al., 2001; Shah et al., 2001; Kawano et al., 2004; Cote et al., 2015). Here, we observed increased nuclear localization of ERα, but not ERβ, during estrus in myenteric neurons in the mouse colon. Nuclear ERα localization results from cytoplasmic receptors being translocated to the nucleus to trigger transcriptional effects via estrogen response elements. The observed increase in nuclear expression of ERα in myenteric neurons during estrus correlates with increased nNOS expression in myenteric neurons and changes in colonic motility. More myenteric neurons were immunoreactive for NOS during estrus compared to proestrus in the mouse distal colon. The percentage of NOS immunoreactive neurons observed during estrus (48 ± 2%) is also greater than that reported for male C57Bl/6 mice [41.7%; (Gamage et al., 2013)] and for male colons of other mouse strains such as Balb/c [approximately 35%; Balb/c (Sang and Young, 1996)].

It is well established that pharmacological blockade of NOS increases CMC frequency (Fida et al., 1997; Powell and Bywater, 2001) suggesting that NO levels in the myenteric plexus exert an inhibitory brake on the CMC pattern generator. Blockade of NOS with NOLA significantly increased the number of CMCs in proestrus female mice but had no effect in estrus females. However, this treatment significantly reduced the resting colonic diameter in both groups suggesting that levels of nNOS in inhibitory motor neurons are not cycle-dependent. An alternative explanation is that there are cycle-dependent changes in nNOS levels in interneurons, which also influence colonic constriction. CMC frequency increases and the corresponding interval between CMCs reduces in response to NOS inhibition in male mice (Lyster et al., 1995; Fida et al., 1997; Powell et al., 2003; Roberts et al., 2007). These findings suggest that during proestrus, where plasma estrogen levels are high but actual genomic effects of estrogens are low, female mice present a similar phenotype to male mice where estrogen levels/effects are generally low. However, during the estrus phase, where functional effects of estrogens are higher, NOS blockade did not eliminate relaxation of colonic smooth muscles. The expression of NOS in a new population of interneurons with unknown connectivity could produce mutually compensating changes in the motor pattern generator leading to no net effect of inhibition of NO synthesis during estrus. This indicates a direction for further experimentation. In contrast, during proestrus, neural NOS levels within the ganglia are low enough that reduced synthesis can disinhibit the pattern generator. It is important to note that NO can suppress synaptic transmission in the ENS (Tamura et al., 1993; Yuan et al., 1995) and NO in fact mediates inhibitory post synaptic potentials (IPSPs) in guinea-pig colon (Dickson et al., 2007).

These data suggest that there is a sexually dimorphic and estrus cycle-dependent change in nNOS expression in myenteric neurons and NO-mediated colonic motility. We show that this effect correlates with the nuclear expression of ERα. Therefore, estrogens are probably responsible for the changes observed. However, it is important to understand the mechanisms that could be responsible for increased numbers of nNOS neurons during estrus, as this occurs in a relatively short time window of only 18–24 h between proestrus and estrus. Further clarification of changes in enteric neuronal proportions should be carried out for a number of additional neurochemical markers and with larger animal numbers in a future study to better characterize changes in enteric neuronal properties in mice during the estrus cycle.

Our findings using an *ex vivo* organ bath approach suggest involvement of nuclear ERα in modulating colonic motility. This method is advantageous in that the gastrointestinal structure remains relatively intact, enabling analysis of the propagating motor complexes under close to physiological conditions. A previous study by Li et al. (2016) using muscle strip preparations reported that estrogens acting on the GPER membrane estrogen receptor are responsible for cyclic changes in gut smooth muscle contraction. This group also showed that application of G1, the selective GPER agonist, increased nNOS protein expression in mouse LMMP preparations (Li et al., 2016). In contrast with our approach, this study did not assess the relative contributions of the nuclear and membrane estrogen receptors in modulating colonic motility. In their experiments, blockade of GPER only partially recovered estrogen-mediated smooth muscle contractions in muscle strips, suggesting that estrogen also triggers contractions via other pathways such as by activation of nuclear estrogen receptors. The study by Li et al. (2016) therefore does not fully support an exclusive role of GPER in modulating estrogen-mediated colonic motility and is compatible with the current findings suggesting that nuclear ERα plays a role in estrogen modulation of colonic motility.

### Possible Mechanisms by Which Estrogen Affects NO

Estrogens can affect gene regulation and posttranscriptional modifications of NOS protein (Grohe et al., 2004; Showkat Ali et al., 2012). Proper functioning of the NOS enzyme depends on several factors including the availability of cofactors and post-translational modifications (Michel and Vanhoutte, 2010). Estrogen elevates the expression of BH_4_, a cofactor binding to NOS which is an important limiting step in NO biosynthesis (Klatt et al., 1993; Vasquez-Vivar et al., 1999; Lam et al., 2006; Serova et al., 2006). *In vivo* experiments conducted with female diabetic rats confirmed that a diabetes-induced delay in gastric emptying can be restored by supplementation of BH_4_ through restoring nNOS activity and NO synthesis (Gangula et al., 2010). Importantly, it has been reported that there are estrogen response elements in the promotor region of the nNOS gene (Chiueh et al., 2003). Therefore, estrogen can modulate NO functioning through directly affecting nNOS gene regulation or via cofactors required for NO biosynthesis, further illustrating the importance of this pathway in the gastrointestinal tract.

In summary, estrogen receptors are expressed in the vast majority of myenteric neurons and increased nuclear expression of ERα was observed alongside an increase in nNOS numbers during estrus in mice. Here we propose that cyclic changes in propagating colonic motility in mice are due to a change in nNOS activity due to genomic effects of estrogen acting on ERα.

## Data Availability Statement

The raw data supporting the conclusions of this article will be made available by the authors, without undue reservation.

## Ethics Statement

The animal study was reviewed and approved by the University of Melbourne Animal Experimentation Ethics Committee.

## Author Contributions

GB and SN performed the experiments. JB and GB conceived and designed the experiments. GB, EH-Y, and JB wrote the manuscript. All authors have approved the final version of the manuscript and agreed to be accountable for all aspects of the work.

## Conflict of Interest

The authors declare that the research was conducted in the absence of any commercial or financial relationships that could be construed as a potential conflict of interest.

## Publisher’s Note

All claims expressed in this article are solely those of the authors and do not necessarily represent those of their affiliated organizations, or those of the publisher, the editors and the reviewers. Any product that may be evaluated in this article, or claim that may be made by its manufacturer, is not guaranteed or endorsed by the publisher.
